# Foraging niche shift maintains breeding parameters of a colonial waterbird during range expansion

**DOI:** 10.1002/ece3.6030

**Published:** 2020-02-07

**Authors:** Charlotte Francesiaz, Elizabeth Yohannes, Aurélien Besnard, Nicolas Sadoul, Thomas Blanchon, Arnaud Béchet

**Affiliations:** ^1^ CNRS EPHE UM SupAgro IRD INRA UMR 5175 CEFE PSL Research University Montpellier France; ^2^ Tour du Valat Institut de recherche pour la conservation des zones humides méditerranéennes Arles France; ^3^ UAM – Equipe Limicoles et oiseaux protégés Réserve de Chanteloup ONCFS‐OFB L'île d'Olonne France; ^4^ Stable Isotope Lab Limnological Institute University of Constance Konstanz Germany; ^5^ Les Amis des Marais du Vigueirat Marais du Vigueirat Arles France

**Keywords:** body condition, *Chroicocephalus genei*, dispersal, isotopic niche, limit of distribution, stable isotopes

## Abstract

Relating the effects of foraging niche variation to reproductive dynamics is critical to understand species response to environmental change. We examined foraging niche variations of the slender‐billed gull (*Chroicocephalus genei*), a nomadic colonial waterbird species during its range expansion along the French Mediterranean coast over a 16‐year period (1998–2013). We investigated whether range expansion was associated with a change in chick diet, breeding success, and chicks body condition. We also examined whether breeding success and chicks body condition were explained by diet and colonial characteristics (number of pairs, laying phenology, habitat, and locality). Diet was characterized using dual‐stable isotopic proxies (*δ* ^13^C and *δ* ^15^N) of feather keratin from 331 individuals subsampled from a total of 4,154 chicks ringed and measured at 18 different colonies. *δ* ^13^C decreased and *δ* ^15^N increased significantly during range expansion suggesting that chicks were fed from preys of increasing trophic level found in the less salty habitat colonized by the end of the study period. Niche shift occurred without significant change of niche width which did not vary among periods, habitats, or localities either. Breeding success and chick body condition showed no consistent trends over years. Breeding success tended to increase with decreasing *δ* ^13^C at the colony level while there was no relationship between stable isotope signatures and chick body condition. Overall, our results suggest that even if range expansion is associated with foraging niche shift toward the colonization of less salty and more brackish habitats, the shift had marginal effect on the breeding parameters of the Slender‐billed gull. Niche width appears as an asset of this species, which likely explains its ability to rapidly colonize new locations.

## Graphical abstract

We examined the change in foraging niche of a colonial waterbird which suddenly enlarge its breeding distribution. In spite of a diet shift, reproductive success and chicks' body condition were only marginally affected. Interestingly, the impressive plasticity of this species allows maintaining its breeding parameters despite important changes of nesting habitat.

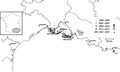

## 1 INTRODUCTION

Global changes have been shown to sharply and rapidly affect the distribution of bird populations (Barnosky et al., 2011; Cahill et al., 2014). These changes may either ease the spread of species or restrain their distribution depending on their ability to track changes in habitat distribution and availability. If species fail to adapt and/or cannot shift their geographic range to track suitable conditions, then they may face disadvantage or exhibit extinction (e.g., Walther et al., 2002). Conversely, climate change has progressively offered new suitable habitats to high‐temperature dwelling species that have in turn gradually increased or shifted their range northward (Devictor, Julliard, Jiguet, & Couvet, 2008). Climate change may also increase preys availability that promote population growth and in turn colonization of new areas by opportunistic species expanding their range (e.g., in yellow‐legged gulls [Duhem, Roche, Vidal, & Tatoni, 2008] or cormorants [Takahashi, Kameda, Kawamura, & Nakajima, 2016]). Finally, some species increase their range after successful introduction of individuals in novel and appropriate habitats (e.g., invasive species; Dukes & Mooney, 1999; Stohlgren & Schnase, 2006). The ability of species to cope with such change of their environments, either by local adaptation or plasticity in resource exploitation, will largely determine their persistence in a fast‐changing world (Toor et al., 2017).

Diagnosing the causes of distribution changes, that is, whether it results from improvement or degradation of the overall status of a species, is a key question for conservation. These distribution shifts may indeed be associated with the evolution of several behavioral processes or traits (Whitney & Gabler, 2008) that may lead to niche shift or expansion or may correspond to simple stochastic successful colonization events of suitable, but previously unused, areas (Oro & Ruxton, 2001). Overall, changes in distribution reflect processes at individual and population levels that remain poorly understood and difficult to investigate in detail (Payo‐Payo et al., 2017). Among the axes of the niche, foraging niche is of crucial importance in case of new environment colonization. Despite the known importance of diet on population dynamics (e.g., on survival [Descamps, Boutin, Berteaux, McAdam, & Gaillard, 2009, Ford, Ellis, Olesiuk, & Balcomb, 2010, Duriez, Ens, Choquet, Pradel, & Klaassen, 2012] and reproduction [Kvarnemo, 1997; Rutz & Bijlsma, 2006; Tavecchia, Pradel, Genovart, & Oro, 2007; Wise, 1979]), how foraging niche shift shapes species distribution during phases of range expansion has poorly been studied (Skórka, Lenda, Martyka, & Tworek, 2009). Both diet shift and range expansion could influence each other. Indeed, range expansion can lead to diet shift as species adapt to novel prey resources available in the newly colonized area. Conversely, diet shift to prey of higher profitability may drive range expansion by favoring the tracking of novel preys in their preferred habitats.

To study the relationship between range expansion and foraging niche, information on both parameters must be available on a large number of individuals. Colonial birds are perfect models to gather information on a large number of individuals, and colonies are often easy to detect which make it simple to follow their distribution.

**Figure 1:**
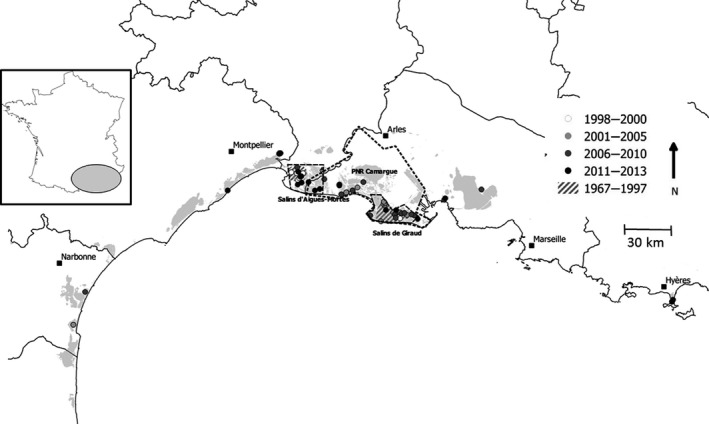
Geographic distribution of the Slender‐billed gulls breeding sites between 1967 and 2013 along the French Mediterranean coast

Here, we studied a highly mobile colonial waterbird, which exhibited spectacular range expansion in the south of France in the last 50 years and explored the relationship between its range expansion and its foraging niche. The Slender‐billed gull, *Chroicocephalus genei*, is a colonial waterbird exhibiting low colony site philopatry (Acker et al., 2017)*.* This species settled in the south of France in the late 1960s and remained confined to the Camargue (Rhône river delta; Figure 1) for thirty years. In the 2000s, the population exhibited an abrupt range expansion with new colonies establishing alongside the eastern and western section of the French Mediterranean coast (Doxa et al., 2013; Simon et al., in prep). Two questions emerged from this expansion pattern: (a) Is this expansion associated with a change in foraging niche? and if so, (b) is the change in foraging niche favorable to the species and lead to an increase in breeding success and chick body condition?

To answer these questions, we explored variations in diet of Slender‐billed gulls over a 16‐year period (1998–2013) in 18 colonies (23 colony‐year) and evaluated if these variations were related to breeding success and body condition of chicks. We used dual‐stable isotopes signatures (carbon *δ* ^13^C and nitrogen *δ* ^15^N) of feathers sampled from 331 chicks as proxies to (a) assess prey nutrient sources (Mizutani, 1990) and to (b) detect possible spatiotemporal variations in the diet and foraging niche of slender‐billed gulls during their range expansion. We then evaluated if chicks body condition was related to variations in stable isotope values and the corresponding isotopic niche width while taking into account (a) habitat type (lagoon vs. saltpans) since the diversity of prey is relatively reduced in saltpans (Britton & Johnson, 1987), (b) seasonal phenology (date of colony settlement) since the availability of prey can vary along the breeding season (Yohannes, Arnaud, & Béchet, 2014), (c) the location of the colony, inside or outside the Camargue, since the protection of the Rhône delta—a Natural Regional Park—may provide resources in higher density, and (d) colony size since it can influence either favorably or negatively the capacity of parental provision to chicks and the number of chicks per pair (Gonzalez‐Solis, Oro, Jover, Ruiz, & Pedrocchi, 1997; Ward & Zahavi, 1973).

## 2 MATERIAL AND METHODS

### 2.1 Study species

The Slender‐billed gull is a colonial bird (Laridae) with a range extending from western India to West Africa (del Hoyo, Elliott, & Sargatal, 1996). Several life history traits of this species suggest it is adapted to unstable and ephemeral habitats such as high breeding dispersal capacities between years and short breeding period associated with crèching behavior allowing them to reduce the time spent on the breeding site (Besnard, Gimenez, & Lebreton, 2002). Slender‐billed gulls generally forage by swimming at the surface of the water (del Hoyo et al., 1996). While they feed on aquatic invertebrates such as brine shrimps (*Artemia* sp.) in saltpans (del Hoyo et al., 1996), they are also known to feed on small fish or scavenge on fish discards following fishermen (e.g., *Atherina*, Snow, Gillmor, & Perrins, 1998) either in freshwater and brackish coastal areas or at sea (e.g., Anchovies or Sardines; Bicknell, Oro, Camphuysen, & Votier, 2013; Cama, Abellana, Christel, Ferrer, & Vieites, 2012; Fasola, Bogliani, Saino, & Canova, 1989).

### 2.2 Population census and distribution change

Exhaustive colony censuses were conducted over the entire French Mediterranean coast since 1967, the earliest record of the first breeding pairs. Before 1973, only one or two pairs were reported in the Camargue and no specific monitoring of the colonies was conducted. Afterward, the species has been censused all along the French Mediterranean coast with annual aerial surveys in the Camargue to quickly detect colonies in vast wetland complexes (the rest of the area, where access is easier, being extensively censused by a network of volunteers). The number of breeding pairs was then recorded by nest counts at the peak of the egg‐laying period, and the number of chicks fledged was estimated by weekly counts of the crèche (Sadoul, 1996). These censuses were first conducted every three years between 1973 and 1991, then annually between 1993 and 2016. No data are available for 1992 and 1996.

From few pairs in the 1970s, the population increased through the 1990s, up to 850 pairs in 2001, and remained roughly at this size afterward (Doxa et al., 2013). From 1967 to 2003, all colonies were located in a relatively small area of c.60 km long. Breeding sites were located within two main areas of the Camargue: the saltpans of Aigues‐Mortes and of Salin‐de‐Giraud (Figure 1). In 2004, a colony was established in the saltpans of Lapalme, 130 km west from the closest previous colonies of Aigues‐Mortes. Grand Bastit and the saltpans of Fos‐sur‐Mer were colonized in 2006, these two sites being at the exterior border of the Camargue. In 2009, a colony set up at the saltpans of Pesquiers, 145 km east from the previous eastern sites of Salin‐de‐Giraud saltpans (Figure 1).

### 2.3 Breeding success, feather sampling, and body measurements

For 16 successive breeding seasons (1998–2013), chicks of a set of colonies were counted and caught just before fledging by herding the crèche into a corral. All chicks captured were ringed and measured (tarsus length to the nearest 1 mm and body weight to the nearest 50 g using a 5‐kg Pesola spring balance). We randomly selected feathers of 8–16 ringed fledglings at one to three colonies each year (23 colony‐year from 18 different colony sites over the study period), in order to explore both interannual and intra‐annual variations in isotope signatures. Overall, we obtained a complete dataset of 4,154 chicks measured (full dataset) from which we extracted a subsample of 331 individuals with isotope data (reduced dataset).

### 2.4 Stable isotope laboratory analysis

Prior to laboratory analysis, feathers were washed in 2:1 chloroform:methanol solution and then rinsed with distilled water. Stable isotope assays were performed on air‐dried and homogenized samples of approximately 0.6 mg that were weighed into tin cups and combusted using Vario Micro cube elemental analyzer (Germany). CO_2_ and N_2_ gases emitted were then analyzed in an interfaced Micromass (Manchester, UK) Isoprime Isotope Ratio Mass Spectrometer (IRMS) with every 7 unknowns separated by two laboratory standards; two sulfanilamides (Iso‐prime internal standards) and two casein standards. All stable isotope ratios are expressed in per mil (‰) using the *δ* notation: *δX* = [(*R*_sample_/*R*_standard_) − 1] × 1,000; where *X* is the ^15^N or ^13^C and *R* is the corresponding ratio of heavy/light (^13^C/^12^C or ^15^N/^14^N) isotope. *R*_standard_ is the ratio of the international references, which for carbon is Vienna Pee Dee belemnite (PDB), and for nitrogen is AIR. Internal laboratory standards indicated measurement errors (*SD*) of ±0.03‰ for *δ* ^13^C, ±0.12‰ for *δ* ^15^N. All isotope analyses were performed at the Limnological Institute, University of Constance, Germany.

### 2.5 Spatiotemporal variations of Slender‐billed gull diet

We distinguished four periods based on the spatial dynamics of the species: (a) 1998–2000 corresponding to a period when all colonies were located in the saltpans of the Camargue, either at Salin‐de‐Giraud or at Aigues‐Mortes; (b) 2001–2005 marked by first colony settlements outside saltpans and high emigration rates outside France (Acker et al., 2017); (c) 2006–2010 corresponding to the return of many individuals back from outside the study area and the subsequent colonization of Grand Bastit and Hyères; (d) 2011–2013 corresponding to the stabilization of a set of colonies outside the Camargue.

We first used linear mixed model (LMM with Gaussian distribution and identity link) to evaluate *δ* ^13^C and *δ* ^15^N variations among the four periods, habitats (lagoon vs. saltpans), location of the colony in/out the Camargue, colony size, and phenology (peak of laying date expressed as the number of days elapsed after the 1st of May). We used colony site nested within year as a random factor. We then used standard ellipse area (SEAc) as a measure of the isotopic niche width of each colony (Jackson et al., 2012; Newsome, del Rio Martinez Rio, Bearhop, & Phillips, 2007). This measure is derived from Bayesian inference and is particularly adapted to small samples and variations of sample size as we have here (Batschelet, 1981; Jackson, Inger, Parnell, & Bearhop, 2011; Syväranta, Lensu, Marjomäki, Oksanen, & Jones, 2013). We tested whether SEAc differed between periods, habitats, location of the colony in/out the Camargue, colony size, and phenology. As SEAc did not follow a Gaussian distribution, we used Kruskal–Wallis tests for the first three analyses and Spearman's rank tests for the two last ones.

### 2.6 Slender‐billed gull colony breeding success

We then explored if—at the colony level—breeding success varied as a function of diet. For this, we first modeled the probability of reproductive failure using a logistic model (binomial distribution of the error term) and then modeled the number of chicks fledged at a colony as a response variable, using the number of breeding pairs (log transformed) as an offset (negative binomial distribution of the error term). We evaluated through model selection if average *δ* ^13^C, average *δ* ^15^N, or SEAc would improve model fit. We found no correlation between our variables (Pearson's correlation: *δ* ^13^C and SEAc, *r* = −.37 [IC95%: −0.67; 0.05]; *δ* ^13^C vs. *δ* ^15^N, *r* = −.4 [IC95%: −0.7; 0.02]; and *δ* ^15^N vs. SEAc, *r* = −.12 [IC95%: −0.051; 0.305]). We also evaluated if breeding success varied along years and periods over the global dataset of all colonies monitored along the French Mediterranean to ascertain that our sample of colonies was representative of the global picture.

### 2.7 Slender‐billed gull chicks' body condition

When estimating body condition, chick tarsus length was used as a body size indicator, which significantly correlates with chicks mass (standardized major axis (SMA) regression of the log‐transformed mass–length relationship; *R* ^2^ = .49; *p* < .0001, slope = 2.48). We used the scaled mass index to estimate chicks' body condition (Peig & Green, 2009, 2010).

Using the full dataset, we examined if chicks body condition varied among periods, between habitats and colony location (in/out the Camargue) using linear mixed models (with Gaussian distribution and identity link) with colony site nested in year as a random factor. We also evaluated if colony average *δ* ^13^C, *δ* ^15^N, SEAc, phenology, and colony size could improve the model fit. We performed the same analysis on the reduced dataset to evaluate the direct effect of individual *δ* ^13^C and *δ* ^15^N feather signature on chicks' body condition.

For both datasets, we included all variables in the analyses and then fitted all possible combinations of variables without interactions. We centered and scaled all explanatory variables prior model fitting. Model comparison was based on the AICc. Model averaging was used to produce estimates and 95% confidence intervals of the effects retained on a set made of models within less than 2 points of AIC of the best model (Burnham & Anderson, 2002).

All analyses were conducted under R environment (R Development Core Team, 2017). More specifically, SEAc was estimated with package SIBER (Stable Isotope Bayesian Ellipse R; Jackson et al., 2011) and linear mixed models were fitted with lme4 package (Bates, Mächler, Bolker, & Walker, 2015). Model comparisons were performed using the “Dredge” function of package MuMIn (Barton, 2015).

### 2.8 Ethics statement

All the conducted field works comply with current laws of France (permit delivered by the Research Center by Ringing Bird Populations, Natural History Museum of Paris; program n°326).

## 3 RESULTS

### 3.1 Spatiotemporal variations of chick diet

**Figure 2:**
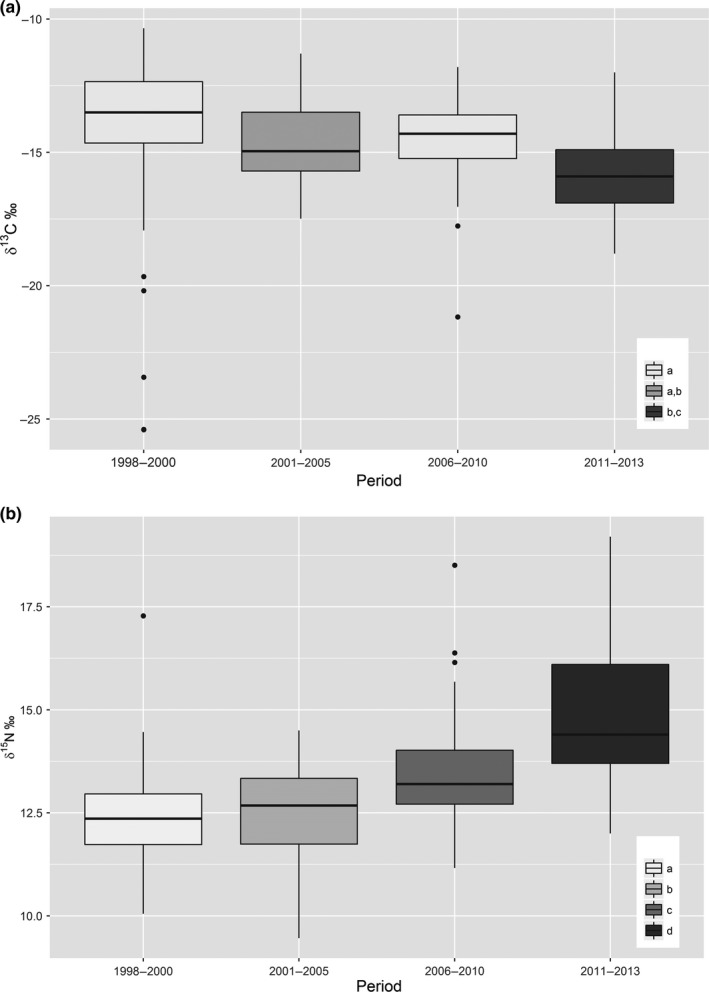
Box plot of the variation of *δ* ^13^C and *δ* ^15^N along the study period with the median and the upper and lower quartiles represented, respectively, by the bold line and the limit of the boxes. Differences between periods are shown by colors and compact letter display as the result of Tukey's post hoc tests (box plot that share the same letters are not significantly different)

Slender‐billed gulls chicks feathers had an overall mean *δ* ^13^C of −14.66‰ (range: −25.40‰; −10.35‰) and of *δ* ^15^N of +13.21‰ (range: +9.46‰; +19.20‰). *δ* ^13^C varied significantly among periods with the second and last periods showing the lowest values (Tukey's post hoc tests, Figure 2a). No other colony characteristics (size, habitat, location, and phenology) were retained after model averaging to explain *δ* ^13^C variations (ESM Table S1a). *δ* ^15^N was lower in saltpans than outside (*β *= −1.42 ± 0.3173) and increased significantly along periods (Tukey's post hoc tests, Figure 2b). No other colony characteristics were retained by model selection to explain *δ* ^15^N variation (ESM Table S1b).

**Figure 3:**
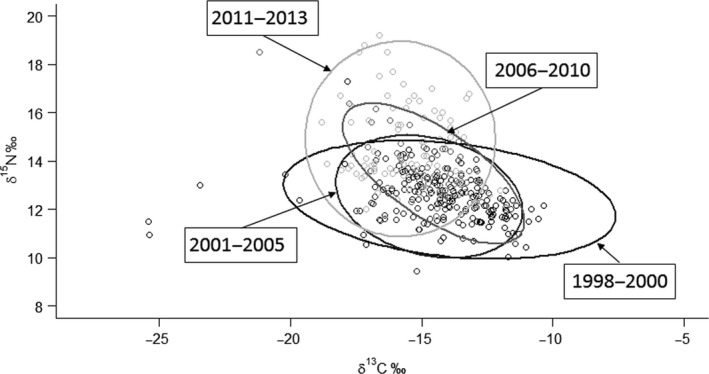
Standard ellipse area (SEAc) of Slender‐billed gull colonies along the four time periods considered. Each dot corresponds to the isotopic signature of an individual. SEAc did not differ significantly from one another

Overall, the standard ellipse area did not differ among periods (Kruskal–Wallis *χ* ^2^ = 4.61, *df* = 3, *p* = .20; Figure 3), neither between habitats (Kruskal–Wallis *χ* ^2^ = 0.96, *df* = 1, *p* = .32), nor between locations in/out the Camargue (Kruskal–Wallis *χ* ^2^ = 1.14, *df* = 1, *p* = .28). Finally, SEAc was not significantly correlated with colony size nor to phenology (Spearman's rank tests *r*_s_ = .24, *p* = .26 and *r*_s _= −.16, *p* = .44, respectively).

### 3.2 Slender‐billed gull breeding success

Among the 79 colonies of the global dataset of Slender‐billed gulls monitored along the French Mediterranean coast from 1998 to 2013, there were 42 cases of complete failure, with no variation of colony failure proportion among periods (*χ* ^2^ = 3.57, *df* = 3, *p* = .31). Whether we considered the global dataset (*N* = 37 colonies without complete failure) or the isotope dataset (23 colonies), model selection indicated no clear temporal trend in breeding success. However, we found a significant lower breeding success in the second period of the study compared with the other periods (*β* = −0.67 ± 0.30 and *β* = −0.46 ± 0.17 for the global and isotope dataset, respectively; ESM, Table S2a,b). Breeding success also increased with decreasing values of the average *δ* ^13^C of the colony (*β* = −0.18 ± 0.04) but was not correlated neither with *δ* ^15^N nor with SEAc (ESM, Table S2b).

### 3.3 Slender‐billed gull chicks' body condition

**Figure 4:**
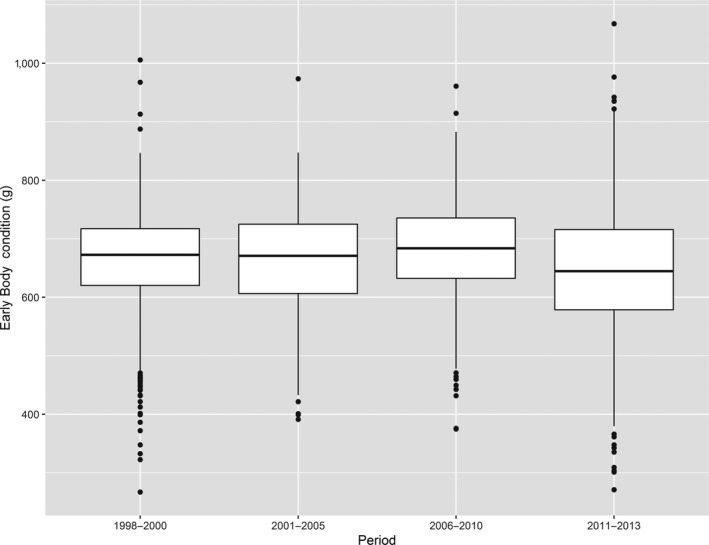
Body condition of chicks (expressed as scaled mass index in g—see Section 2) among the four periods. We found no difference between time periods during the range expansion of the Slender‐billed gull

Chicks body condition did not vary significantly between periods (Figure 4), but there were sometimes quite important differences among colonies of the same year (ESM Figure S2), suggesting that some colonies might benefit from better foraging areas. Model selection did not retain any covariable significantly correlated with body condition variations among chicks either on the full or on the isotope dataset (ESM Tables S3 and S4).

## 4 DISCUSSION

Our results show that the range expansion of the Slender‐billed gull that occurred over 1998–2013 was accompanied by a shift in foraging niche used by breeding birds. While *δ* ^15^N values in chicks' dietary sources increased over the entire study period (particularly in 2011), *δ* ^13^C values decreased in the second and last period of range expansion. Yet, despite the changes in diet detected, we found no significant change in niche width (SEAc). Only lower *δ* ^13^C, associated with less saline waters (Ramirez et al., 2012; Rendon, Rendon‐Martos, Garrido, & Amat, 2011; Yohannes et al., 2014), translated in improvement of colony breeding success suggesting possible provisioning constraints in saltpans.

The shift in foraging niche appears to be associated with the gulls expanding their range into a new habitat, that is, colonizing the less salty habitat of the lagoons but it remains unclear whether dispersal caused diet change or the other way around. Because high breeding dispersal rate is a well‐documented evolutionary strategy for the Slender‐billed gull (Besnard, 2001), the observed range expansion might only be the result of this behavior which has consequently forced gulls to change their diet accordingly. If Slender‐billed gull colony choice is mainly determined by factors linked to breeding island intrinsic qualities regarding terrestrial predator avoidance, diet flexibility is an asset to adapt to the need to track the best nesting conditions. High interannual colony dynamics of this species observed in very stable habitats like saltpans makes it a probable process. In this case, it would be range expansion that would have forced gulls to adapt their diet. Alternately, despite their interest for predator protection, saltpans offer preys of lower profitability (because fish like *Atherina *sp. cannot maintain themselves above 50 g/L) than more brackish areas so that Slender‐billed gull breeding success may be constrained in such habitat. In this case, range expansion may have been favored by increased profitability of preys in the new habitats. Slender‐billed gulls are generally observed wandering in small groups all along the Mediterranean coast before stopping to settling at only a few colony sites (unpublished data). This prospection time may be used as a mean to evaluate foraging habitat quality. Prospection has been showed to play a key role in site selection in other colonial gulls (Boulinier, Danchin, Monnat, Doutrelant, & Cadiou, 1996; Ponchon et al., 2015).

### 4.1 Spatiotemporal diet variation despite isotopic niche width upholding

Isotopic signatures from chick feathers of Slender‐billed gull found in our study were generally on a par with those reported from Spain where gulls used saltpans, natural marshes but also fish farms: −14.66‰ versus −16.62‰ for *δ* ^13^C and 13.21‰ versus 13.12‰ for *δ* ^15^N (Ramirez et al., 2012). Here, isotopic signatures suggest temporal changes in diet concomitant with the geographic spread of gull colonies along the French Mediterranean coast. The decreasing *δ* ^13^C values also suggest that birds tended to feed in less saline waters over years (Yohannes et al., 2014), which is in accordance with the spread of French colonies from saltpans to lagoons and more brackish habitats. The temporal trend of *δ* ^15^N is in accordance with the change of habitats since prey composition differs between saltpans and less salty habitats. Indeed, the increased of *δ* ^15^N during the study period suggests an increase in prey trophic level used by adults to feed their chicks (Peterson & Fry, 1987). In our case, using prey isotopic signatures from the study of the species in Spain (Ramirez et al., 2012), it likely means that along the study period Slender‐billed gulls foraged in an increasing proportion of fish preys (e.g., *Atherina boyeri*) available in lagoons and more brackish habitats relative to brine shrimps (*Artemia *spp.) only found in saltpans. In its eastern core areas, fish composed half of its diet which is also composed on marine insects captured in the mud and plant materials (del Hoyo et al., 1996; Snow, Gillmor, & Perrins, 1998). The shift of foraging niche appears thus associated with the colonization of brackish wetlands which correspond to a preference for fish diet as described in their core areas.

However, the lack of a clear association between colony location and foraging niche parameters and width suggests that change in nesting habitat does not necessarily translate in change of foraging habitat. Indeed, in some years, we found high signatures of *δ* ^15^N in chicks raised in high salinity ponds of saltpans suggesting that adults were able to get prey items from the surrounding brackish canals or wetlands, typically characterized with higher *δ* ^15^N values, such as fish (Ramirez et al., 2012). For example in 2011, the value of *δ* ^15^N was very high even if over 3 sites, and 2 were situated in saltpans. This distinction between foraging and breeding areas is well‐known in colonial birds (Gibbs & Kinkel, 1997) and seems to concern the Slender‐billed gull as well. Interestingly, these high values of *δ* ^15^N were associated with successful reproduction in each colony in 2011. There must be an advantage of foraging further to find high‐quality preys even when breeding in saltpans (where fish cannot survive).

The wide isotopic niche breadth of the Slender‐billed gull could be explained either by the co‐occurrence of different individual foraging strategies or by the individual foraging shift along the breeding season. Tracking individual foraging strategies along chick provisioning with GPS devices could allow discriminating between these two hypotheses.

### 4.2 Influence of environment on breeding success and chick body condition

Interestingly, a lower breeding success was found during the second period of the study marked with high emigration rate outside the south of France and first colony settlements outside saltpans (Acker et al., 2017). However, Slender‐billed gulls were able to maintain their breeding parameters during the consecutive periods of range expansion despite a noticeable change in diet. Indeed, both breeding success and chicks' body condition remained stable across periods afterward.

Given the likely higher profitability of preys used by gulls at the end of the study period as shown by *δ* ^15^N isotopic variations, this raises the question of the reasons for the lack of apparent benefit for productivity. Since quality and abundance of preys are two side of the same coin to explain foraging ecology (Stephens & Krebs, 1986; Wright, Both, Cotton, & Bryant, 1998), there may exist a trade‐off between prey profitability and prey availability in our system. Indeed, in saltpans, Artemia spp constitute low profitability but abundant, easy to catch and highly predictable resource during the breeding season as the salinity which drives its abundance is optimized annually by dedicated management aimed at salt production (Paracuellos et al., 2002). On the contrary, while in lagoons gulls may find preys of higher trophic levels such as fish, their availability (or catchability) may be lower. The potential quality‐availability trade‐off observed in our study has also been shown in pigeon guillemots (Litzow, Piatt, Abookire, & Robards, 2004) another species foraging on unpredictable patchy environment.

### 4.3 Slender‐billed gull range expansion

Slender‐billed gulls first stayed confined to the Camargue area until 2004 when the population size sharply increased. Then from 2004 to 2015, colonies started to spread over both the West and the East of the Camargue to extend progressively along about 250 km of coastline (Figure 1). However, this large spread happened when breeding population size remained relatively stable (Doxa et al., 2013). Our results do not support the hypothesis that improvement of breeding success was the main driver of large‐scale dispersal. Although improved breeding success was not detectable, the availability of suitable food in lagoons could have assisted slender‐billed gulls dispersal by maintaining their reproductive effort/output.

Range expansion may then have resulted from other processes such as a behavioral adaptation to habitat unpredictability. Indeed, the Slender‐billed gull present low site tenacity as shown for the population breeding in southern France (Acker et al., 2017). Its ability to change colony site from one year to another is likely an adaptation to environmental stochasticity (Sanz‐Aguilar et al., 2014). Yet, the scale at which this colony displacements occur is not clearly described in the literature. In France, colony locations have changed almost every year at the local scale but individual birds show a relatively strong philopatry at the French Mediterranean coast scale (Acker et al., 2017). Hence, when the habitat is good enough, Slender‐billed gull may try to breed in the same ecological patch from year to years even if they change exact location of colony site every year (for other reason such as being less predictable for predators for instance; Besnard et al., 2002).

### 4.4 Potential evolutionary responses to global change

Current climate change scenarios predict increasing temperatures, changes in precipitation, drought, and sea‐level rise, but also altered hydrological cycles, including wetland salinization, a major constraint for waterbirds (Masero et al., 2017). A key challenge for species facing these changes will be their ability to cope up with the rapid rate of changes either by plasticity or by evolutionary response. Our results provide support that foraging niche width is advantageous to exploit changing habitats and decrease the physiological constraints imposed by high salinity. However, further research on the effect of diet change on other life history parameters (e.g., survival) is required to assess possible evolutionary responses to such changes (Radchuk et al., 2019).

### 4.5 Perspectives

Isotopic approach proved to be useful in unveiling diet variations in the Slender‐billed gull. However, the behavioral components of niche width remain unknown. For now, we cannot reject the hypothesis where birds moved for whatever reason (evolution strategy, disturbance at previous sites, avoidance of predation, etc.) and eat according to the availability of preys around the new site. Diet shift may thus only be a by‐product of dispersal. Foraging distances, habitat use, and the relative role of individual and population variations in niche width would deserve further investigation. Defining home range size would help to further understand the link between foraging choices and population dynamics and eventually support efficient conservation measures. Indeed, coupling isotopic studies with adult tracking during the provisioning period using modern GPS tracking devices (e.g., Veen, Dallmeijer, Schlaich, Veen, & Mullié, 2019) could provide a better understanding of how species will adjust their effort to environmental variations in order to maintain their fitness.

## CONFLICT OF INTEREST

None declared.

## AUTHORS CONTRIBUTION

N. Sadoul initiated the long‐term monitoring and capture–ringing–resighting of the Slender‐billed gull in the Camargue. N. Sadoul and T. Blanchon collected the data and coordinated the fieldwork with many volunteers. E.Yohannes conducted the isotopic analyses and contributed to the writing of the papers and the interpretation of the results. C. Francesiaz, A. Besnard, and A. Béchet performed the analysis and wrote the paper.

## Data Availability

The data that support the findings of this study are openly available in dryad https://doi.org/10.5061/dryad.g79cnp5m1.

## References

[ece36030-bib-0001] Acker, P. , Francesiaz, C. , Béchet, A. , Sadoul, N. , Lessells, C. M. , Pijl, A. S. , & Besnard, A. (2017). Insights on dispersal and recruitment paradigms: Sex‐ and age‐dependent variations in a nomadic breeder. Oecologia, 186(1), 85–97. 10.1007/s00442-017-3972-7 29063200

[ece36030-bib-0002] Barnosky, A. D. , Matzke, N. , Tomiya, S. , Wogan, G. O. U. , Swartz, B. , Quental, T. B. , … Ferrer, E. A. (2011). Has the Earth's sixth mass extinction already arrived? Nature, 471, 51–57. 10.1038/nature09678 21368823

[ece36030-bib-0003] Barton, K. (2015). MuMIn-Model selection and model averaging based on information criteria (AICc and alike). R package version 1.15.1.

[ece36030-bib-0004] Bates, D. , Mächler, M. , Bolker, B. M. , & Walker, S. C. (2015). Fitting linear mixed‐effects models using lme4. Journal of Statistical Software, 67, 1–48.

[ece36030-bib-0005] Batschelet, E. (1981). Circular statistics in biology. London, New York: Academic Press. ISBN: 9780120810505 0120810506.

[ece36030-bib-0006] Besnard, A. (2001). Evolution de l'élevage des poussins en crèche chez les laridés.

[ece36030-bib-0007] Besnard, A. , Gimenez, O. , & Lebreton, J.‐D. (2002). A model for the evolution of crèching behaviour in gulls. Evolutionary Ecology, 16, 489–503. 10.1023/A:1020809528816

[ece36030-bib-0008] Bicknell, A. W. , Oro, D. , Camphuysen, K. C. , & Votier, S. C. (2013). Potential consequences of discard reform for seabird communities. Journal of Applied Ecology, 50, 649–658. 10.1111/1365-2664.12072

[ece36030-bib-0009] Boulinier, T. , Danchin, E. , Monnat, J. , Doutrelant, C. , & Cadiou, B. (1996). Timing of prospecting and the value of information in a colonial breeding bird Timing of prospecting and the value of information in a colonial breeding bird. Journal of Avian Biology, 27, 252–256. 10.2307/3677230

[ece36030-bib-0010] Britton, R. H. , & Johnson, A. R. (1987). An ecological account of a Mediterranean Salina – the Salin‐De‐Giraud, Camargue (S. France). Biological Conservation, 42, 185–230.

[ece36030-bib-0011] Burnham, K. P. , & Anderson, D. R. (2002). A practical information-theoretic approach. Model selection and multimodel inference (2nd ed.). New York, NY: Springer.

[ece36030-bib-0012] Cahill, A. E. , Aiello‐Lammens, M. E. , Fisher‐Reid, M. C. , Hua, X. , Karanewsky, C. J. , Ryu, H. Y. , … Wiens, J. J. (2014). Causes of warm‐edge range limits: Systematic review, proximate factors and implications for climate change. Journal of Biogeography, 41, 429–442.

[ece36030-bib-0013] Cama, A. , Abellana, R. , Christel, I. , Ferrer, X. , & Vieites, D. (2012). Moving to the sea: A challenge for an inshore species, the slender‐billed gull. Marine Ecology Progress Series, 463, 285–295.

[ece36030-bib-0014] del Hoyo, J. D. , Elliott, A. , & Sargatal, J. (1996). Handbook of the birds of the world. Volume 3: Hoatzin to Auks. Barcelona, Spain: Lynx Edicions.

[ece36030-bib-0015] Descamps, S. , Boutin, S. , Berteaux, D. , McAdam, A. G. , & Gaillard, J.‐M. (2009). Cohort effects in red squirrels: The influence of density, food abundance and temperature on future survival and reproductive success. Journal of Animal Ecology, 78, 182–190. 18179549 10.1111/j.1365-2656.2007.01340.x

[ece36030-bib-0016] Devictor, V. , Julliard, R. , Jiguet, F. , & Couvet, D. (2008). Birds are tracking climate warming, but not fast enough. Proceedings of the Royal Society B: Biological Sciences, 275, 2743–2748. 10.1098/rspb.2008.0878 PMC2605823 18713715

[ece36030-bib-0017] Doxa, A. , Besnard, A. , Bechet, A. , Pin, C. , Lebreton, J.‐D. , & Sadoul, N. (2013). Inferring dispersal dynamics from local population demographic modelling: The case of the slender‐billed gull in France. Animal Conservation, 16, 684–693. 10.1111/acv.12048

[ece36030-bib-0018] Duhem, C. , Roche, P. , Vidal, E. , & Tatoni, T. (2008). Effects of anthropogenic food resources on yellow‐legged gull colony size on Mediterranean islands. Population Ecology, 50, 91–100. 10.1007/s10144-007-0059-z

[ece36030-bib-0019] Dukes, J. S. , & Mooney, H. A. (1999). Does global change increase the success of biological invaders? Trends in Ecology & Evolution, 14, 135–139. 10.1016/S0169-5347(98)01554-7 10322518

[ece36030-bib-0020] Duriez, O. , Ens, B. J. , Choquet, R. , Pradel, R. , & Klaassen, M. (2012). Comparing the seasonal survival of resident and migratory oystercatchers: Carry‐over effects of habitat quality and weather conditions. Oikos, 121, 862–873. 10.1111/j.1600-0706.2012.20326.x

[ece36030-bib-0021] Fasola, M. , Bogliani, G. , Saino, N. , & Canova, L. (1989). Foraging, feeding and time‐activity niches of eight species of breeding seabirds in the coastal wetlands of the Adriatic Sea. Italian Journal of Zoology, 56, 61–72.

[ece36030-bib-0022] Ford, J. K. B. , Ellis, G. M. , Olesiuk, P. F. , & Balcomb, K. C. (2010). Linking killer whale survival and prey abundance: Food limitation in the oceans' apex predator? Biology Letters, 6, 139–142. 10.1098/rsbl.2009.0468 19755531 PMC2817236

[ece36030-bib-0023] Gibbs, J. P. , & Kinkel, L. K. (1997). Determinants of the size and location of great blue heron colonies. Colonial Waterbirds, 20, 1–7. 10.2307/1521757

[ece36030-bib-0024] Gonzalez‐Solis, J. , Oro, D. , Jover, L. , Ruiz, X. , & Pedrocchi, V. (1997). Trophic niche width and overlap of two sympatric gulls in the southwestern mediterranean. Oecologia, 112, 75–80. 10.1007/s004420050285 28307378

[ece36030-bib-0025] Jackson, A. L. , Inger, R. , Parnell, A. C. , & Bearhop, S. (2011). Comparing isotopic niche widths among and within communities: SIBER ‐ Stable Isotope Bayesian Ellipses in R. Journal of Animal Ecology, 80, 595–602. 10.1111/j.1365-2656.2011.01806.x 21401589

[ece36030-bib-0026] Jackson, M. C. , Donohue, I. , Jackson, A. L. , Britton, J. R. , Harper, D. M. , & Grey, J. (2012). Population‐level metrics of trophic structure based on stable isotopes and their application to invasion ecology. PLoS ONE, 7, 1–12. 10.1371/journal.pone.0031757 PMC3283663 22363724

[ece36030-bib-0027] Kvarnemo, C. (1997). Food affects the potential reproductive rates of sand goby females but not of males. Behavioral Ecology, 8, 605–611. 10.1093/beheco/8.6.605

[ece36030-bib-0028] Litzow, M. A. , Piatt, J. F. , Abookire, A. A. , & Robards, M. D. (2004). Energy density and variability in abundance of pigeon guillemot prey: Support for the quality-variability trade-off hypothesis. Journal of Animal Ecology, 73, 1149–1156.

[ece36030-bib-0029] Masero, J. A. , Abad‐Gómez, J. M. , Gutiérrez, J. S. , Santiago‐Quesada, F. , Senner, N. R. , Sánchez‐Guzmán, J. M. , … Villegas, A. (2017). Wetland salinity induces sex‐dependent carry‐over effects on the individual performance of a long‐distance migrant. Scientific Reports, 7(1), 6867. 28761120 10.1038/s41598-017-07258-w PMC5537338

[ece36030-bib-0030] Mizutani, H. (1990). Carbon isotope ratio of feathers reveals feeding behavior of cormorants. The Auk, 107(2), 400–403.

[ece36030-bib-0031] Newsome, S. D. , del Rio Martinez, C. , Bearhop, S. , & Phillips, D. L. (2007). A niche for isotope ecology. Frontiers in Ecology and the Environment, 5, 429–436.

[ece36030-bib-0032] Oro, D. , & Ruxton, G. D. (2001). The formation and growth of seabird colonies: Audouin's gull as a case study. Journal of Animal Ecology, 102, 374–384.

[ece36030-bib-0033] Paracuellos, M. , Castro, H. , Nevado, J. C. , Ona, J. A. , Matamala, J. J. , Garcia, L. , & Salas, G. (2002). Repercussions of the abandonment of Mediterranean saltpans on waterbird communities. Waterbirds, 25, 492–498. 10.1675/1524-4695(2002)025[0492:ROTAOM]2.0.CO;2

[ece36030-bib-0034] Payo‐Payo, A. , Genovart, M. , Sanz‐Aguilar, A. , Greño, J. L. , García‐Tarrasón, M. , Bertolero, A. , … Oro, D. (2017). Colonisation in social species: The importance of breeding experience for dispersal in overcoming information barriers. Scientific Reports, 7, 42866 10.1038/srep42866 28211483 PMC5314353

[ece36030-bib-0035] Peig, J. , & Green, A. J. (2009). New perspectives for estimating body condition from mass/length data: The scaled mass index as an alternative method. Oikos, 118, 1883–1891.

[ece36030-bib-0036] Peig, J. , & Green, A. J. (2010). The paradigm of body condition: A critical reappraisal of current methods based on mass and length. Functional Ecology, 24, 1323–1332.

[ece36030-bib-0037] Peterson, B. J. , & Fry, B. (1987). Stable isotopes in ecosystem studies. Annual Review of Ecology and Systematics, 18, 293–320. 10.1146/annurev.es.18.110187.001453

[ece36030-bib-0038] Ponchon, A. , Chambert, T. , Lobato, E. , Tveraa, T. , Grémillet, D. , & Boulinier, T. (2015). Breeding failure induces large scale prospecting movements in the black‐legged kittiwake. Journal of Experimental Marine Biology and Ecology, 473, 138–145. 10.1016/j.jembe.2015.08.013

[ece36030-bib-0039] R Development Core Team (2017). R: A language and environment for statistical computing. Vienna, Austria: R Foundation for Statistical Computing.

[ece36030-bib-0040] Radchuk, V. , Reed, T. , Teplitsky, C. , van de Pol, M. , Charmantier, A. , Hassall, C. , … Kramer‐Schadt, S. (2019). Adaptive responses of animals to climate change are most likely insufficient. Nature Communications, 10, 1–14. 10.1038/s41467-019-10924-4 PMC6650445 31337752

[ece36030-bib-0041] Ramirez, F. , Navarro, J. , Afan, I. , Hobson, K. A. , Delgado, A. , & Forero, M. G. (2012). Adapting to a changing world: Unraveling the role of man‐made habitats as alternative feeding areas for slender‐billed gull (*Chroicocephalus genei*). PLoS ONE, 7(10), e47551. 23094062 10.1371/journal.pone.0047551 PMC3477125

[ece36030-bib-0042] Rendon, M. A. , Rendon‐Martos, M. , Garrido, A. , & Amat, J. A. (2011). Greater flamingos *Phoenicopterus roseus* are partial capital breeders. Journal of Avian Biology, 42, 210–213. 10.1111/j.1600-048X.2011.05236.x

[ece36030-bib-0043] Rutz, C. , & Bijlsma, R. G. (2006). Food‐limitation in a generalist predator. Proceedings of the Royal Society B: Biological Sciences, 273, 2069–2076. 10.1098/rspb.2006.3507 PMC1635485 16846915

[ece36030-bib-0044] Sadoul, N. (1996). Dynamique spatiale et temporelle des colonies de Charadriiformes dans les salins de Camargue: Implications pour la conservation. PhD thesis - Université des Sciences et Techniques Montpellier 2.

[ece36030-bib-0045] Sanz‐Aguilar, A. , Tavecchia, G. , Afan, I. , Ramirez, F. , Doxa, A. , Bertolero, A. , … Oro, D. (2014). Living on the edge: Demography of the Slender‐billed gull in the Western Mediterranean. PLoS ONE, 9, e92674 10.1371/journal.pone.0092674 24664115 PMC3963922

[ece36030-bib-0046] Simon, J. , Gimenez, O. , Sadoul, N. , Doxa, A. , Pradel, R. , Béchet, A. , & Besnard, A. (in prep). Do massive breeding failures drive massive breeding dispersal at local and large scale?

[ece36030-bib-0047] Skórka, P. , Lenda, M. , Martyka, R. , & Tworek, S. (2009). The use of metapopulation and optimal foraging theories to predict movement and foraging decisions of mobile animals in heterogeneous landscapes. Landscape Ecology, 24, 599–609. 10.1007/s10980-009-9333-0

[ece36030-bib-0048] Snow, D. W. , Gillmor, R. , & Perrins, C. M. (1998). The birds of the Western Palearctic: Non-passerines. Oxford, UK: Oxford University Press.

[ece36030-bib-0049] Stephens, D. W. , & Krebs, J. R. (1986). Foraging theory (p. 261). Princeton, NJ: Princeton University Press.

[ece36030-bib-0050] Stohlgren, T. J. , & Schnase, J. L. (2006). Risk analysis for biological hazards: What we need to know about invasive species. Risk Analysis, 26, 163–173. 10.1111/j.1539-6924.2006.00707.x 16492190

[ece36030-bib-0051] Syväranta, J. , Lensu, A. , Marjomäki, T. J. , Oksanen, S. , & Jones, R. I. (2013). An empirical evaluation of the utility of convex hull and standard ellipse areas for assessing population niche widths from stable isotope data. PLoS ONE, 8, 1–8. 10.1371/journal.pone.0056094 PMC3566058 23405254

[ece36030-bib-0052] Takahashi, T. , Kameda, K. , Kawamura, M. , & Nakajima, T. (2016). Food habits of great cormorant Phalacrocorax carbo hanedae at Lake Biwa, Japan, with special reference to ayu *Plecoglossus altivelis altivelis* . Fisheries Science, 72, 477–484.

[ece36030-bib-0053] Tavecchia, G. , Pradel, R. , Genovart, M. , & Oro, D. (2007). Density‐dependent parameters and demographic equilibrium in open populations. Oikos, 116, 1481–1492. 10.1111/j.0030-1299.2007.15791.x

[ece36030-bib-0054] van Toor, M. L. , Arriero, E. , Holland, R. A. , Huttunen, M. J. , Juvaste, R. , Müller, I. , … Safi, K. (2017). Flexibility of habitat use in novel environments: Insights from a translocation experiment with lesser black‐backed gulls. Royal Society Open Science, 4, 160164 10.1098/rsos.160164 28280543 PMC5319309

[ece36030-bib-0055] Veen, J. , Dallmeijer, H. , Schlaich, A. E. , Veen, T. , & Mullié, W. C. (2019). Diet and foraging range of slender‐billed gulls *Chroicocephalus genei* breeding in the Saloum Delta, Senegal. Ardea, 107, 33 10.5253/arde.v107i1.a8

[ece36030-bib-0056] Walther, G.‐R. , Post, E. , Convey, P. , Menzel, A. , Parmesan, C. , Beebee, T. J. C. , … Bairlein, F. (2002). Ecological responses to recent climate change. Nature, 416, 389–395. 10.1038/416389a 11919621

[ece36030-bib-0057] Ward, P. , & Zahavi, A. (1973). The importance of certain assemblages of birds as “information‐centres” for food‐finding. Ibis, 115, 517–534.

[ece36030-bib-0058] Whitney, K. D. , & Gabler, C. A. (2008). Rapid evolution in introduced species, “invasive traits” and recipient communities: Challenges for predicting invasive potential. Diversity and Distributions, 14, 569–580.

[ece36030-bib-0059] Wise, D. H. (1979). Effects of an experimental increase in prey abundance upon the reproductive rates of Two Orb‐Weaving Spider Species (Araneae: Araneidae). Oecologia, 300, 289–300. 10.1007/BF00377433 28309766

[ece36030-bib-0060] Wright, J. , Both, C. , Cotton, P. A. , & Bryant, D. (1998). Quality vs. quantity: Energetic and nutritional trade-offs in parental provisioning strategies. Journal of Animal Ecology, 67, 620–634.

[ece36030-bib-0061] Yohannes, E. , Arnaud, A. , & Béchet, A. (2014). Tracking variations in wetland use by breeding flamingos using stable isotope signatures of feather and blood. Estuarine, Coastal and Shelf Science, 136, 11–18. 10.1016/j.ecss.2013.11.010

