# Supplementary material for: Foraging niche shift maintains breeding parameters of a colonial waterbird during range expansion
Source: Ecol Evol. 2020 Feb 7;10(4):1988–97. doi: 10.1002/ece3.6030 (PMC7042741; doi:10.1002/ece3.6030)
Supplement: Supplementary file 1 [file ECE3-10-1988-s001.docx]

**Electronic Supplementary material**

Table S1. Model selection for the variation of a) δ13C and b) δ15N signature in the chicks’ feathers of Slender-billed gulls fledged in southern France from 1998 to 2013 (N=331) as a function of the four time periods considered (Period), Colony size (Colsize), phenology (Pheno), the location in/out Camargue (Cam), or in/out saltpans (Saltpans). Only models within 2 points of ΔAIC of the best model are presented.

| 1. Model for δ^13^C | k | AICc | ΔAIC | AICω |
| --- | --- | --- | --- | --- |
| Period+ColSize+Pheno | 6 | 1312.2 | 0.00 | 0.120 |
| Cam+Period+ColSize+Pheno | 7 | 1312.3 | 0.04 | 0.118 |
| Period | 4 | 1312.3 | 0.04 | 0.118 |
| Period+ColSize+Pheno+Saltpan | 7 | 1313.0 | 0.73 | 0.084 |
| Cam+Period+ColSize+Pheno+Saltpan | 8 | 1313.5 | 1.27 | 0.064 |
| Period+ColSize | 5 | 1313.6 | 1.36 | 0.061 |
| Cam | 2 | 1313.7 | 1.48 | 0.057 |
| Period+Saltpan | 5 | 1313.9 | 1.64 | 0.053 |
| Period+Pheno | 5 | 1313.9 | 1.65 | 0.053 |
| Period+Cam | 5 | 1314.1 | 1.83 | 0.048 |
|  |  |  |  |  |
| 1. Model for δ^15^N | **k** | **AICc** | **ΔAIC** | **AICω** |
| Period+Saltpan | 5 | 956.4 | 0.00 | 0.301 |
| Period+Pheno+Saltpan | 4 | 956.9 | 0.27 | 0.231 |
| Camargue+Period+Saltpan | 6 | 958.1 | 1.71 | 0.128 |
|  |  |  |  |  |

Table S2. a) Model selection for the variation of the number of chicks fledged in southern France from 1998 to 2013 as a function of Period and Year in the global dataset of 37 colonies without complete failure. b) Model selection for the variation of the number of chicks fledged in southern France from 1998 to 2013 as a function of Period, niche width (SEAc), δ^13^C and δ^15^N of chick feathers in the isotope dataset of 23 colonies. The number of breeding pairs was kept as an offset in all models so that selected variables explain colony breeding success. Only models within 2 points of ΔAIC of the best model are presented.

| 1. Full dataset |  |  |  |  |  |
| --- | --- | --- | --- | --- | --- |
| Model for Breeding success | k | AICc | ΔAIC | AICω |  |
| Period | 3 | 2233.1 | 0.00 | 0.8 |  |
| Period+Year | 4 | 2235.9 | 2.77 | 0.2 |  |
|  |  |  |  |  |  |
| 1. Isotope dataset |  |  |  |  |  |
| Model for Breeding success | k | AICc | ΔAIC | AICω |  |
| δ^13^C+Period | 2 | 1418.1 | 0.00 | 0.440 |  |
| Period+ SEAc | 3 | 1419.6 | 1.44 | 0.214 |  |
|  |  |  |  |  |  |

Table S3. Model selection of the body condition of Slender-billed gulls (standard body mass index) variation as a function of δ^13^C, δ^15^N, niche width (SEAc), the four time periods considered (Period), Colony size (Colsize), phenology (Pheno), the location in/out Camargue (Cam), or in/out saltpans (Saltpans). Only models within 2 points of ΔAIC of the best model are presented.

| Full dataset |  |  |  |  |  |
| --- | --- | --- | --- | --- | --- |
| Model for early body condition | k | AICc | ΔAIC | AICω |  |
| Saltpans | 2 | 48363.4 | 0.00 | 0.068 |  |
| Intercept only | 1 | 48363.6 | 0.17 | 0.062 |  |
| δ^15^N | 2 | 48363.7 | 0.28 | 0.059 |  |
| δ^15^N +Saltpans | 3 | 48364.7 | 1.24 | 0.036 |  |
| Saltpans + SEA | 3 | 48364.8 | 1.40 | 0.034 |  |
| SEA | 2 | 48365.0 | 1.53 | 0.031 |  |
| δ^15^N + Camargue | 3 | 48365.3 | 1.57 | 0.031 |  |
| δ^15^N +SEA | 3 | 48365.3 | 1.86 | 0.027 |  |
| Camargue + Saltpans | 3 | 48365.3 | 1.87 | 0.027 |  |
| Phenology + Saltpans | 3 | 48365.3 | 1.88 | 0.026 |  |
| δ^13^C | 2 | 48365.4 | 1.99 | 0.025 |  |
|  |  |  |  |  |  |
| Isotope dataset |  |  |  |  |  |
| Model for early body condition | k | AICc | ΔAIC | AICω |  |
| Intercept only | 1 | 3710.4 | 0.00 | 0.141 |  |
| Saltpans | 2 | 3711.3 | 0.98 | 0.086 |  |
| Phenology | 2 | 3712.0 | 1.62 | 0.063 |  |
| Phenology +Saltpans | 3 | 3712.2 | 1.83 | 0.056 |  |
| Camargue | 4 | 3712.3 | 1.90 | 0.054 |  |
| δ^13^C | 2 | 3712.3 | 1.91 | 0.054 |  |
| δ^15^N | 2 | 3712.3 | 1.91 | 0.054 |  |
| Colony size | 2 | 3712.3 | 1.92 | 0.054 |  |

Table S4. Estimates ± Standard error, 95% confidence interval and relative importance of the parameters resulting from the averaging of models aimed at explaining body condition from the full and isotope dataset.

| **Full dataset** |  |  |  |  |
| --- | --- | --- | --- | --- |
| Parameters explaining body condition | Estimate ± SE | 2.5% lower CI | 97.5 % upper CI | AICw |
| Saltpans | -10.47 ± 7.72 | -25.61 | 4.65 | 0.46 |
| Colony average δ^15^N | 9.98 ± 8.04 | -5.77 | 25.75 | 0.40 |
| SEAc | -7.55 ± 8.84 | -24.81 | 9.78 | 0.30 |
| Camargue | 6.30 ± 9.57 | -12.47 | 25.07 | 0.26 |
| Phenology | 0.76 ± 8.04 | -15.01 | 16.53 | 0.17 |
| Colony size | 0.97 ± 9.91 | -18.45 | 20.40 | 0.17 |
| Colony average δ^13^C | -2.37 ± 8.09 | -18.25 | 13.50 | 0.21 |
|  |  |  |  |  |
| **Isotope dataset** |  |  |  |  |
| Parameters explaining body condition | Estimate ± SE | 2.5% lower CI | 97.5 % upper CI | AICw |
| Saltpans | -11.95 ± 10.21 | -31.9 | 8.05 | 0.36 |
| Phenology | 9.25 ± 10.99 | -12.3 | 30.7 | 0.30 |
| Camargue | - 3.15 ± 10.52 | -23.7 | 17.4 | 0.19 |
| δ^13^C | -2.12 ± 5.74 | -13.3 | 9.1 | 0.19 |
| δ^15^N | -3.39 ± 7.10 | -17.3 | 10.5 | 0.20 |
| Colony size | -2.57 ± 10.43 | -22.9 | 17.8 | 0.19 |
|  |  |  |  |  |

*
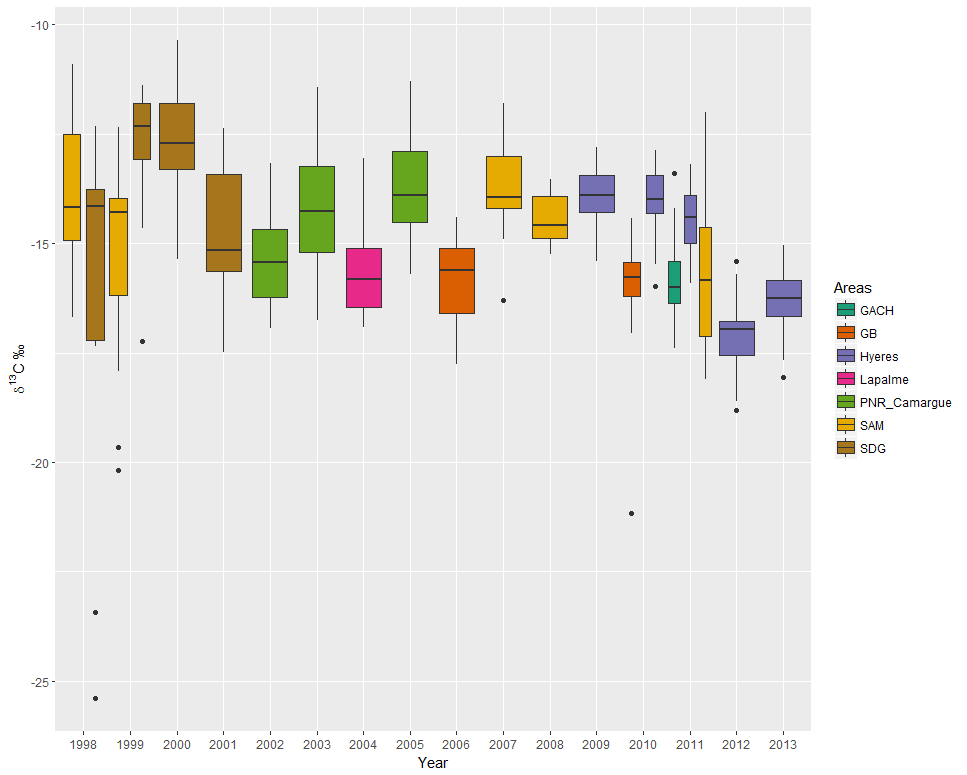
*

*
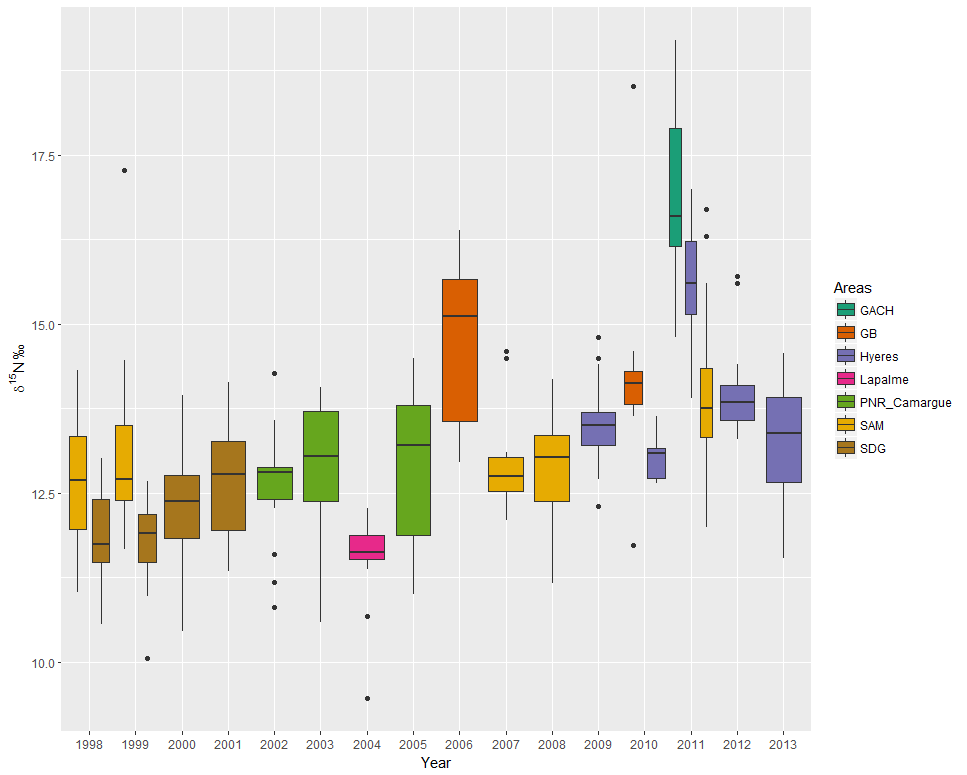
*

*
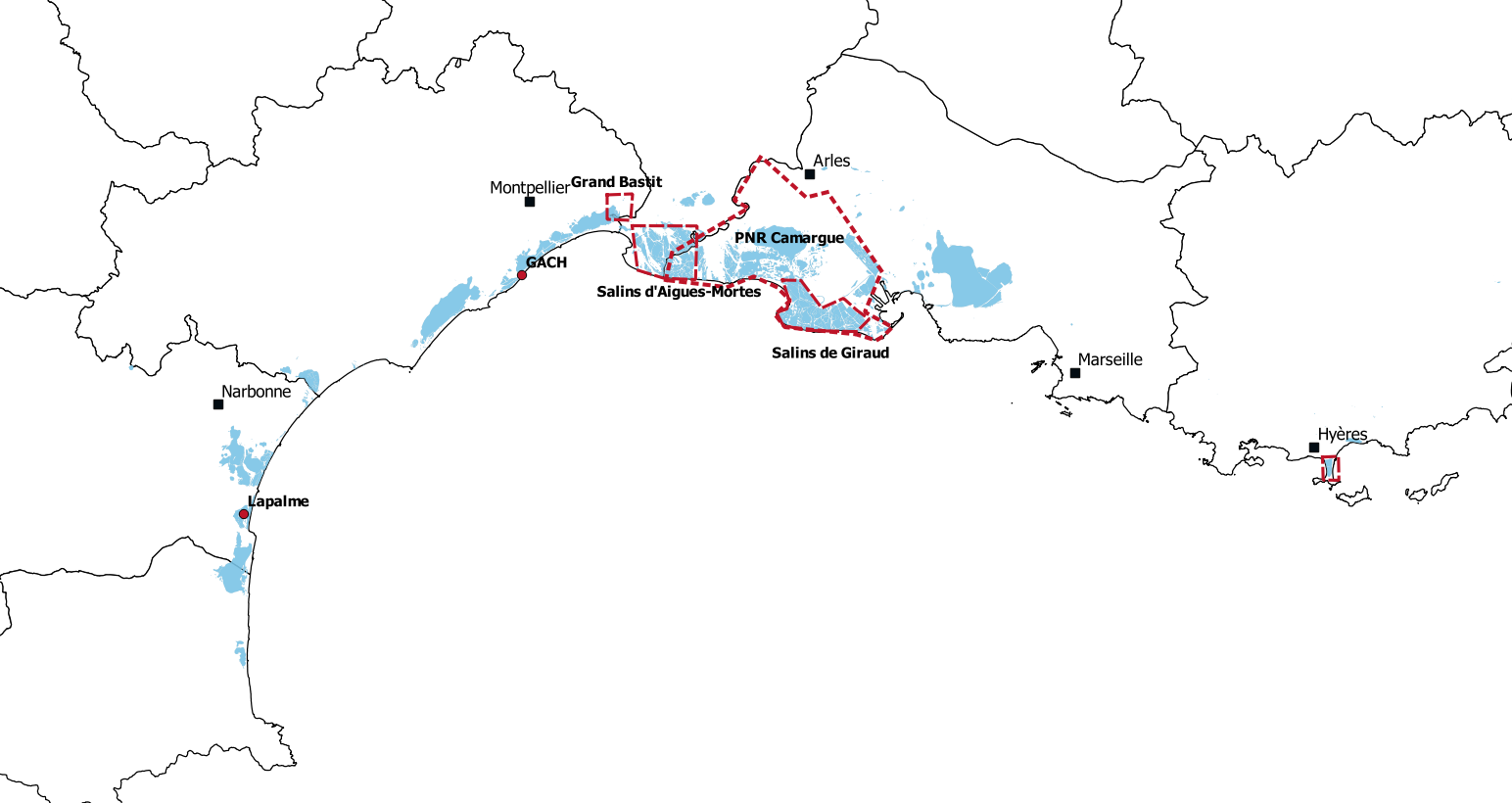
*Figure S1. Boxplots of the variations of δ13C and δ15N by colony for the Slender-billed gulls chicks in the south of France from 1998 to 2013. The map helps finding the regions associated to the categorical names in the boxplots.


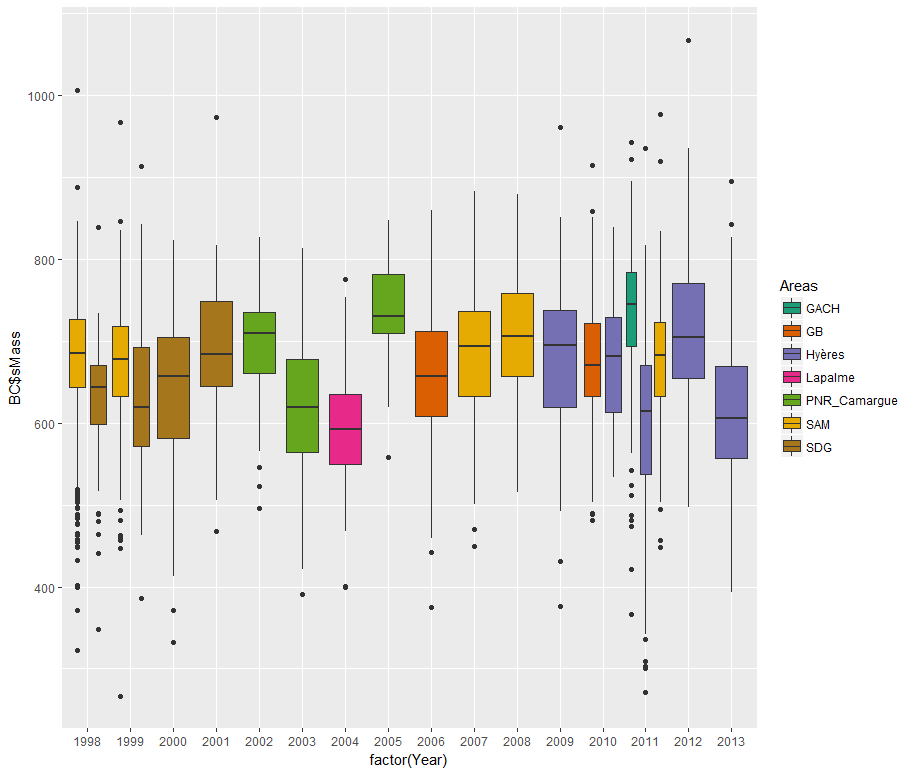


Figure S2. Early body condition of Slender-billed gull chicks in the South of France from 1998 to 2013.


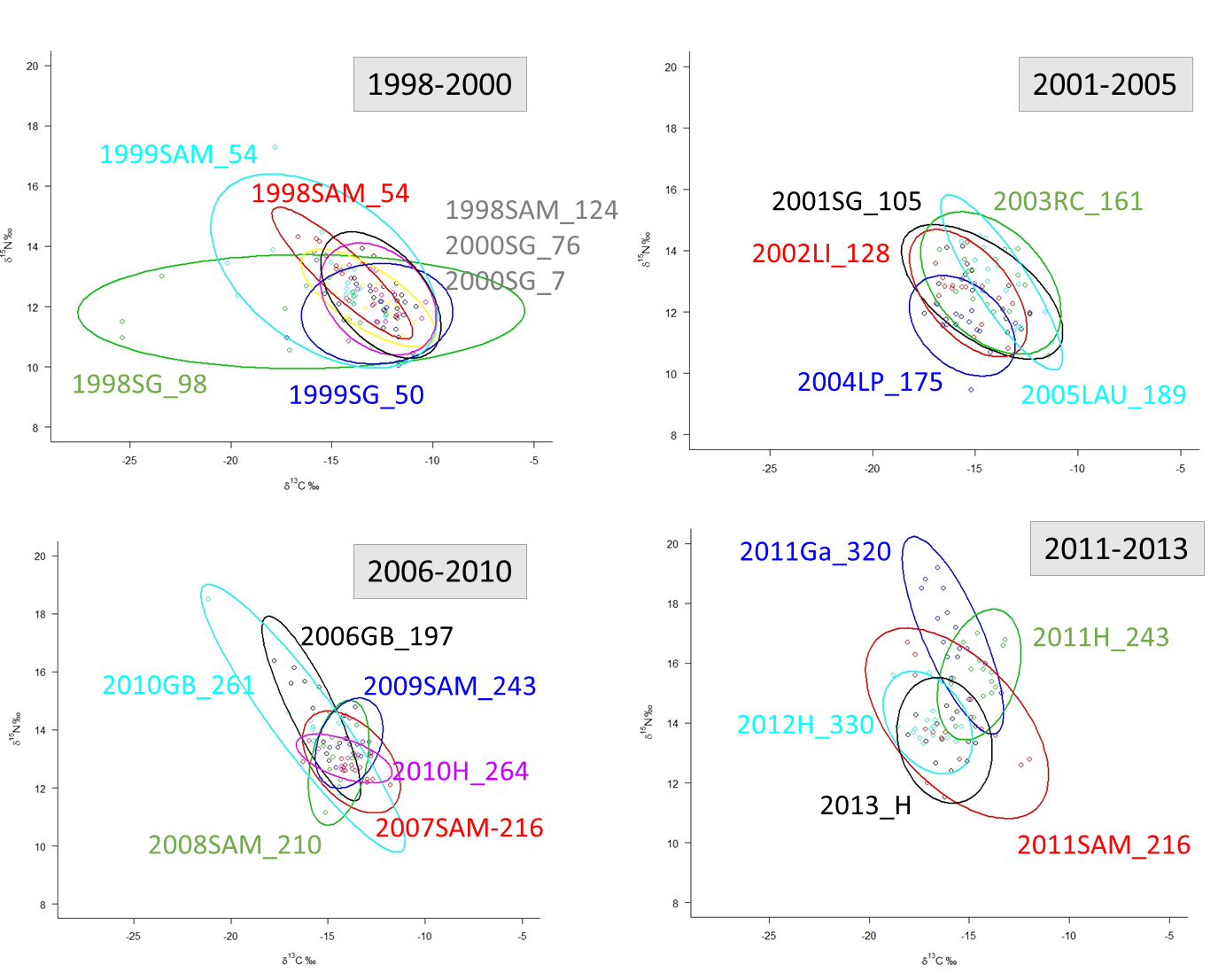


Figure S3. Niche width variation among periods as measured by standard ellipse area (SEAc) for Slender-billed gulls chicks diet in the south of France (1998-2013). Each ellipse represents a colony.


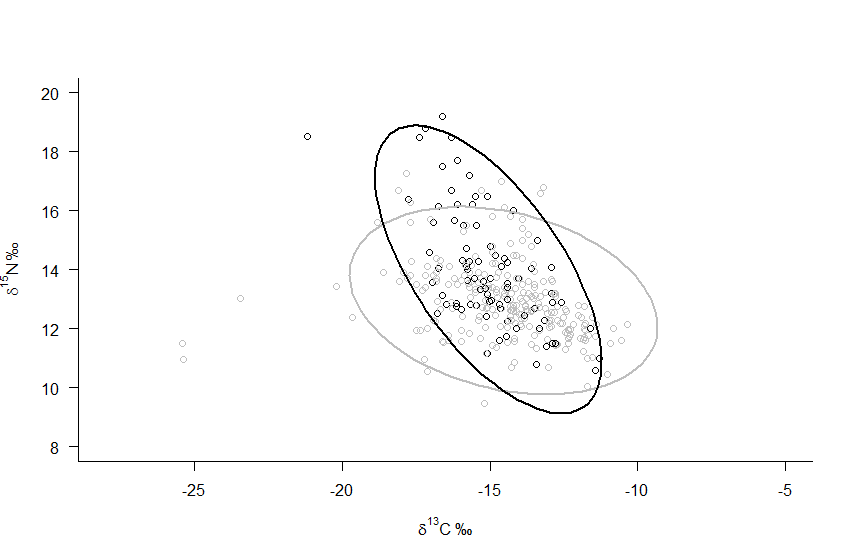
 Figure S4. Niche width as measured by Standard ellipse area (SEAc) of Slender-billed gulls chicks as a function of the chicks being from a colony located in a saltpan or not. Colonies in saltpans are in grey and outside saltpans in black.


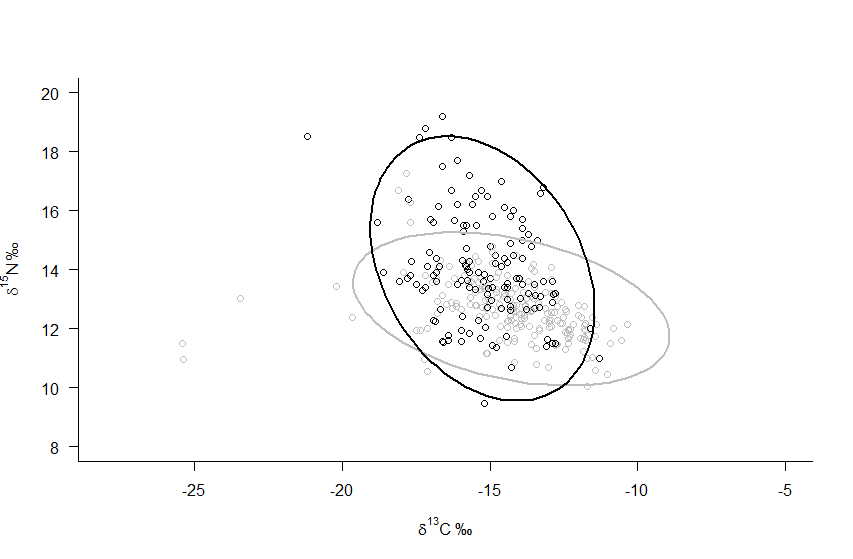


Figure S5. Niche width as measured by Standard ellipse area (SEAc) of Slender-billed gulls chicks as a function of the chicks being from a colony located in the Camargue or not. Colonies in Camargue are in grey and outside saltpans in black.
